# Supplementary material for: Hybrid plasmonic nano-emitters with controlled single quantum emitter positioning on the local excitation field
Source: Nat Commun. 2020 Jul 8;11:3414. doi: 10.1038/s41467-020-17248-8 (PMC7343831; doi:10.1038/s41467-020-17248-8)
Supplement: Supplementary file 1 — Supplementary Information [file 41467_2020_17248_MOESM1_ESM.pdf]

Supplementary information for

Hybrid plasmonic nano-emitters with controlled single quantum emitter positioning on the local excitation field

Ge et al.

Supplementary Note 1. Geometry of the hybrid nanocube as a function of the incident dose used for plasmonic 2-photon polymerization

SEM analysis of hybrid nanosystems based on gold nanocube was carried out for different incident doses. For high incident doses, it is easy to distinguish the polymer on the top and bottom faces. When the dose decreases, the transition area between these two layers become misty, and because the bottom face has larger polymerization action, the peripheral contour of polymer under SEM is assigned to the polymer on the bottom face.

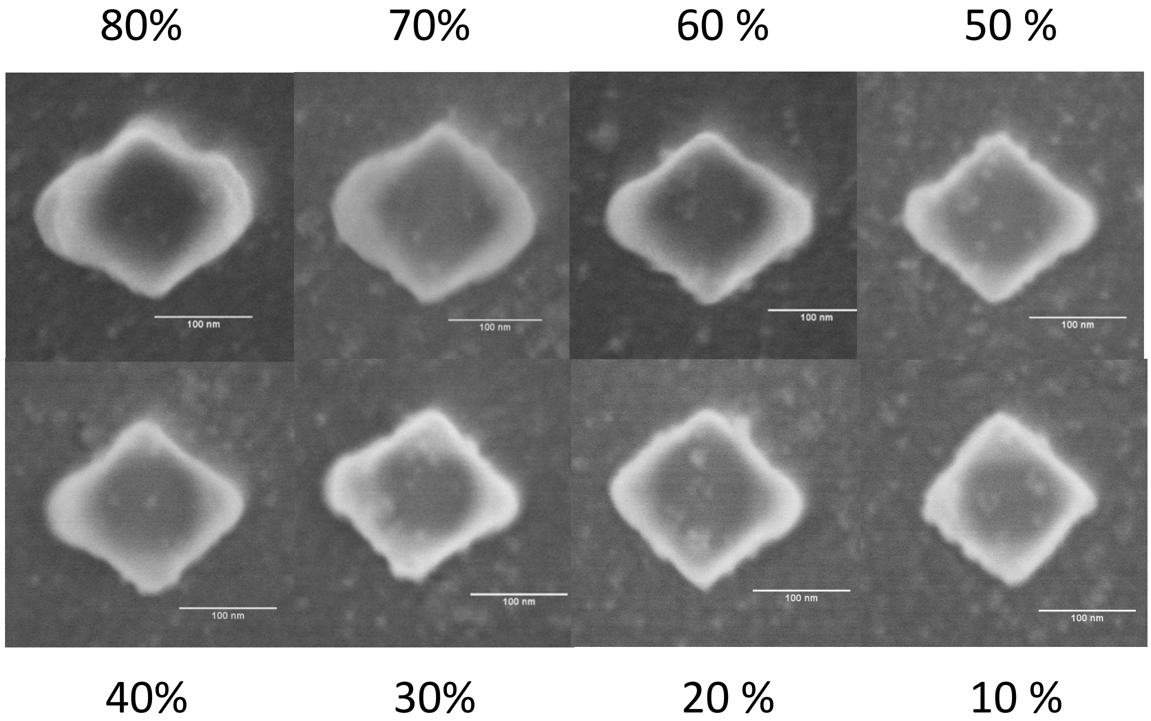

Supplementary Fig. 1 SEM raw images of hybrid nanosystems based on gold nanocubes. The hybrid nano-objects were obtained by plasmon-induced 2-photon polymerization with different incident doses (from 10 to 80 % of the bulk energy polymerization threshold). Objects are different from one image to the other but the nanocubes are identical to each other (see Fig. 1).

Supplementary Note 2. Proof of presence of QDs within the polymerized patterns

Presence of QDs within both formulation and polymerized material is ascertained by three different methods illustrated in Supplementary Fig. 2.

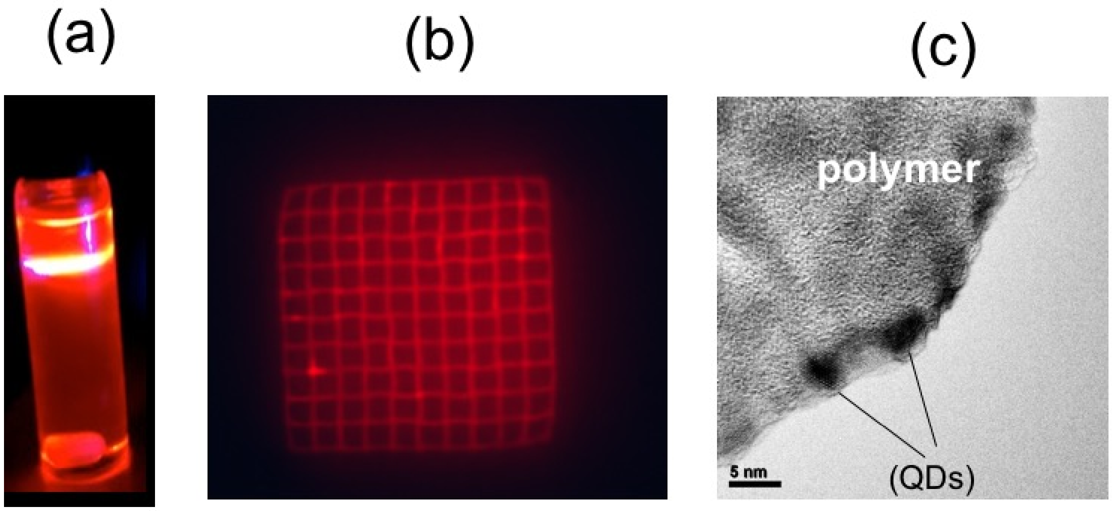

Supplementary Fig. 2 Presence of light-emitting red QDs within formulation and polymer (a) PL signal of the photopolymerizable liquid formulation containing QDs that are attached to PETA monomers, observed under UV illumination. (b) PL image from a polymerized 50 µm wide pattern illuminated with UV light. (c) TEM image taken at the edge of a polymerized drop of formulation containing QDs.

For small thicknesses of the polymer matrix, QDs can be observed at the periphery of the polymerized drops by transmission electron microscopy (TEM), see supplementary Fig. 2c. For polymer material thicknesses > 10 nm, the QDs are hardly discernible on TEM images. This observation allowed us to roughly access the volume density of QDs within the polymer. Supplementary Fig. 2c reveals that the QDs are separated from each other with a distance of about 7 nm. Each 6.7-nm size QDs (see methods) would thus occupy a sphere of about 14 nm in diameter, resulting in a maximum volume density of QDs ~ $7x10^5.\mu m^{-3}$ = $7x10^{-4}$ $QD.nm^{-3}$. A typical integrated polymer lobe has a volume of about $70x25x50$ nm^3 (e. g. Fig. 3a) corresponding to a few tens of QDs per lobe.

Supplementary Note 3. Analysis of the polymer elongation as a function of the incident dose

The probability of 2-photon absorption and resulting polymerization depends quadratically on the local light irradiance I (intensity per surface unit).

$$\mathrm{P} = \mathrm{F}(\mathrm{I}^2) \qquad (1)$$

Function *F* contains all of the processes related to the 2-photon polymerization and is a monotonically increasing function. This photopolymerizable chemical system exhibits a threshold power I_{th}, below which no polymerization can occur. I_{th} is first experimentally accessed by far-field experiments. Photopolymerization depends on both incident light intensity I_{in} and local near-field enhancement f. The latter one locally amplifies the former one. The near-field intensity is supposed to be evanescent. In the case of local

photopolymerization along the nanocube diagonal, in the vicinity of the gold nanocube the local effective irradiance for polymerization is:

$$I_{eff} = f_{max}\, I_{in} \exp(-y/\delta) \qquad (2)$$

Where f_{max} is the maximum intensity enhancement factor inside the local surface plasmon resonance LSPR trigged area, δ represents the LSPR's characteristic intensity decay length, and y is the distance from the nanocube surface along the diagonal direction. Polymerization is achieved only where $I_{eff} > I_{th}$, resulting in the following condition:

$$y < \delta . \ln\left(f_{max} \times \frac{I_{in}}{I_{th}}\right) = y_{max} = \delta .\ \ln(p \times f_{max}) \qquad (3)$$

y_{max} is the observable parameter that can be accessed by SEM or AFM. It corresponds to the polymer elongation represented in Fig. 2e, and Supplementary Figure 3a as a reminder. Equation (3) explains the log-like function shown in Figure 2e. Equation (3) can be rewritten:

$$\frac{1}{p} = f_{max} \times \exp(-y_{max}/\delta) \qquad (4)$$

It should be stressed that 1/p represents the plasmon-induced intensity gain. For example, the fact that the polymer was obtained for p = 0.1 demonstrates an intensity enhancement of, at least, 10. Equation (4) explains the exponential-like function shown in Supplementary Fig. 3b. Analytical fitting leads to the determination of f_{max} and δ. In more detail, in the case of Supplementary Fig. 3b, this leads to f_{max} = 56 and δ = 7 nm. Thus, this method constitutes a unique way of measuring the evanescent decay, the size of the optical nanosource and the plasmon intensity enhancement factor.

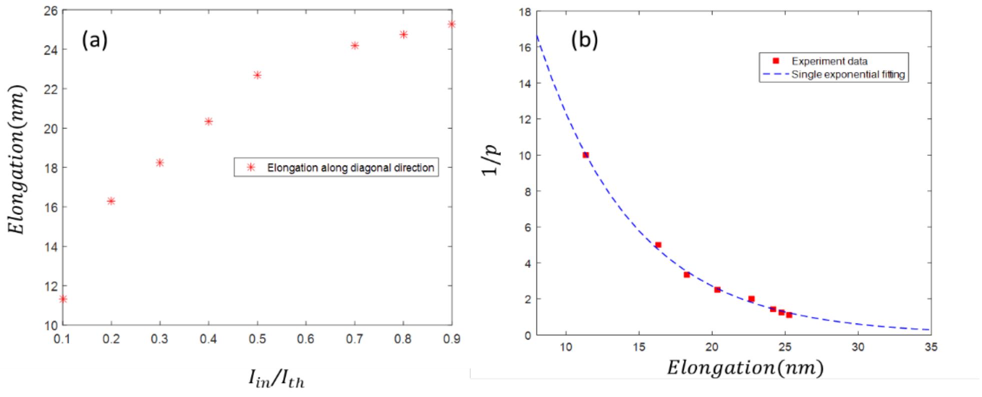

Supplementary Fig. 3. Quantitative analysis of polymer elongation as a function of the incident laser irradiance. (a) Effect of the relative exposure power $p = I_{in}/I_{th}$ on the elongation of the polymerized volume along the nanocube diagonal. (b) Experimental data points (red square) of the $1/p$ ratio plotted as a function of the polymer elongation and exponential fit with a single exponential according to (4).

For comparison, by FDTD calculation we got numerical values f_{max} = 57 and δ = 12 nm respectively. These values compare well with those extracted from the experimental data fit, showing that the method is suitable to the quantification of single gold nanocubes. More

precisely, the FDTD simulation points to a slightly longer decay length δ along spatial position. This difference can be due to the fact that we consider a stable and uniform dielectric environment around the cube for FDTD simulation. During polymerization, the experienced refractive index is expected to change, which could result in a different decay length.

Supplementary Note 4. Differential AFM imaging of the hybrid plasmonic nanosources

In order to further analyze the integrated polymer parts, AFM images were taken before and after local photopolymerization, for incident polarization parallel to a nanocube diagonal. Tapping mode AFM (Bruker Dimension Icon) was used with silicon tips (70 kHz resonance, 1 Hz scan rate). Supplementary Fig. 4a shows the AFM image of a bare gold nanocube on glass substrate. Supplementary Fig. 4b shows the AFM image of precisely the same nanocube after plasmonic 2-photon polymerization (incident dose = 50% threshold dose). Supplementary Figure 4c is the differential AFM resulting from subtraction of Supplementary Fig 4a from Supplementary Fig. 4b. Supplementary Fig. 4d is a cross section profile from 4c along the diagonal. It turns out that the whole nanocube vertical edge got covered with polymer whose height (about 130 nm) is even slightly larger than the nanocube height (127 nm). The slight asymmetry is attributed to a possible asymmetry of the AFM tip shaft and dynamics along the fast scan axis. This asymmetry is also visible in Supplementary Fig. 4a.

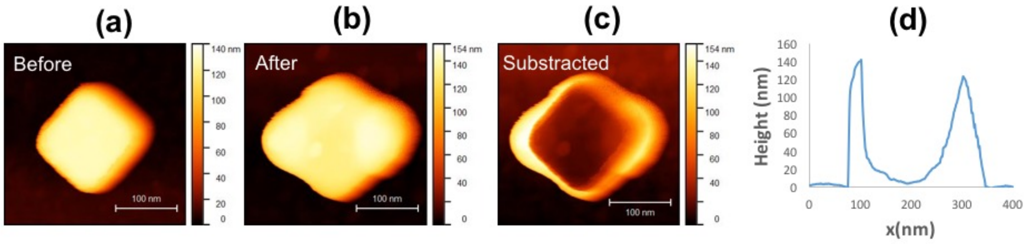

Supplementary Fig. 4 Differential AFM imaging of hybrid nanostructure based on Au nanocube. (a) AFM image of the bare Au nanocube on glass. (b) AFM image of precisely the same nanocube after plasmon induced 2-photon polymerization (c) differential AFM image: (b) minus (a). (d) profile of Fig. (c) along the diagonal.

Supplementary Note 5. Photoluminescence spectrum: comparison between hybrid nanocubes with or without light emitting quantum dot within the polymer lobes

Two kinds of hybrid nanostructures based on gold nanocubes were produced. The first kind contains quantum emitters as described in the article (especially Fig. 2a). As far as the second kind is concerned, no QDs were added into the photosensitive formulation, resulting in a hybrid polymer/gold nanocubes which does not contain any nano-emitters. Photoluminescence spectra were measured on the two kinds of hybrid nanoparticles. The first kind shows clear red spectrum from the CdSe/CdS/Zn QDs (see Supplementary Fig

5a). This spectral signature is not visible on the second kind (Supplementary Fig. 5b), proving that the red spectrum corresponds to the photoluminescence of the QDs.

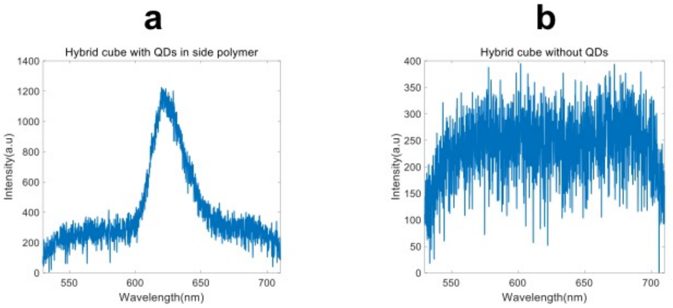

Supplementary Fig. 5 Photoluminescence spectrum from two kinds of single nanocube-based hybrid nanoparticle. (a) case of hybrid nanoparticles whose polymer lobes contain CdSe/CdS/Zn QDs. (b) case of hybrid nanoparticles whose polymer lobes do not contain any QDs.

Supplementary Note 6. Time stability of hybrid plasmonic nano-sources

Four nanocube-based hybrid plasmonic nanosources, identical to that of Fig. 3, were fabricated in order to access the time stability of the PL emission. 405-nm wavelength 25 $\mu W.\mu m^{-2}$ incident power was used (polarization parallel to the QD-containing polymer lobes) except for the fourth one (70 $\mu W.\mu m^{-2}$). Each hybrid nanosource was shined every day for 30 min during a period ranging from 10 to 22 days. PL data (intensity and spectrum) were got randomly. Supplementary Fig. 6 presents these collected data. For 25 $\mu W.\mu m^{-2}$ PL emission stays reasonably stable for five days: % of decrease of the PL intensity is in the 4%-45%. For 70 $\mu W.\mu m^{-2}$, PL intensity quickly drops to 65% of the initial intensity and stays pretty stable for 10 days. Further studies will allow us to investigate the origin and process of photodegradation for the most fragile hybrid nanosources.

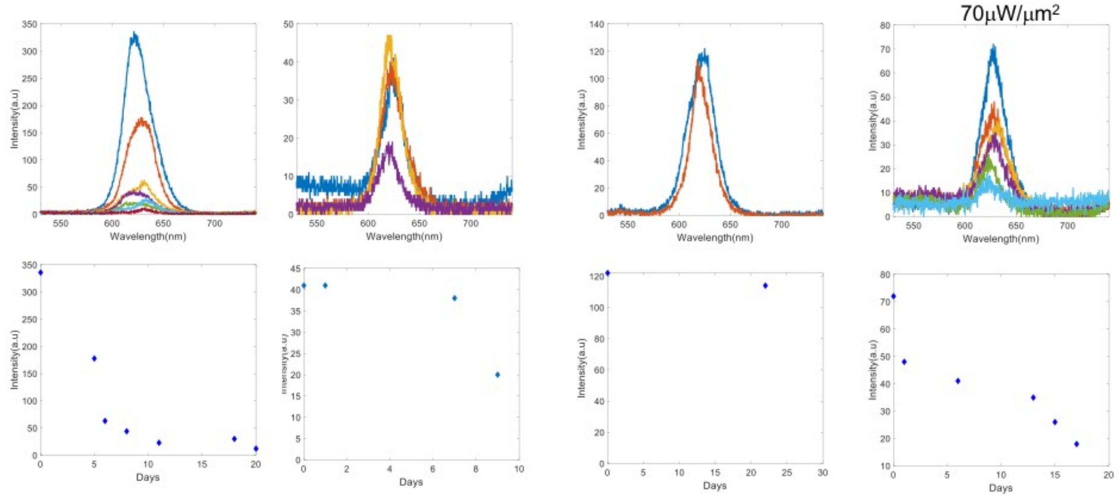

Supplementary Fig. 6 Study of time stability of the photoluminescence from four different hybrid nano-emitters. Each emitter was shined every day for 30 minutes (405 nm incident wavelength) with an incident power of 25 $\mu W.\mu m^{-2}$, except for the last one (70 $\mu W.\mu m^{-2}$).

Supplementary Note 7. PL images from hybrid plasmonic nanosources as a function of the incident angle of polarization

In the case of hybrid nanocubes with polymerized lobes along one diagonal, PL images were acquired for different incident polarization directions to ascertain QD anisotropic spatial distribution. Supplementary Fig. 7 shows a clear tuning of the PL intensity.

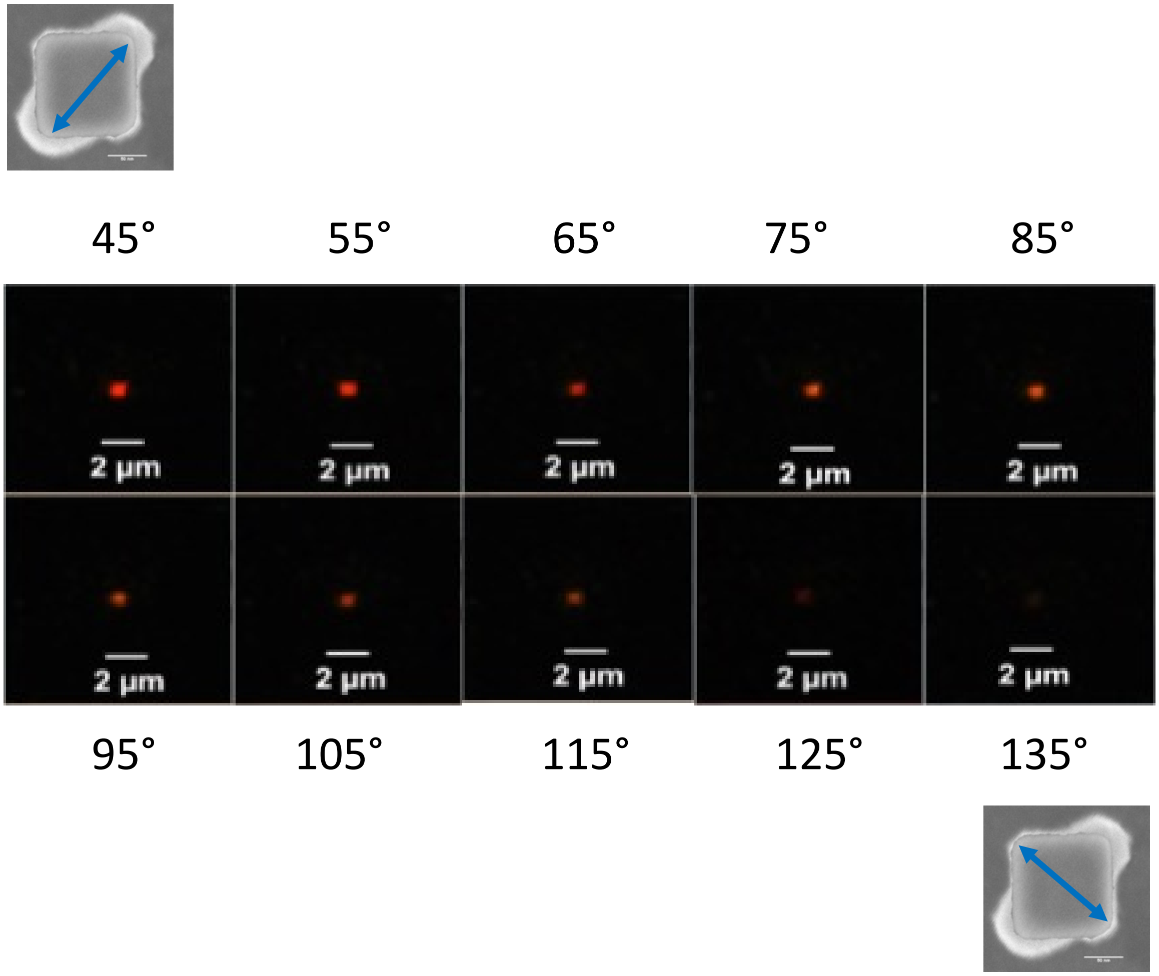

Supplementary Fig. 7 PL image of a hybrid nanosource based of Au nanocube for different incident polarization angles (excitation wavelength λ = 405 nm). Top: polarization angle from 45° to 85° (10° increments). Bottom: polarization angles from 95° to 135° (10° increment) Brightest image (top left): polarization aligned on the polymer lobe axis. Darkest image (bottom right): polarization perpendicular to the polymer lobe axis. The blue arrow represents the direction of the incident polarization.

Supplementary Note 8. Analysis of single QDs in polymer without plasmonic nanoparticles

Supplementary figure 8 shows the schematic diagram of our system used for lifetime and $g^{(2)}$ measurement in the single-photon regime. We used a driven pulsed laser, whose repetition frequency can be modified from 80 MHz to 2.5 MHz. We used 20 MHz for $g^{(2)}$ and 5 MHz for lifetime. The total Instrument Response Function (IRF) of the system

includes all the components having a time response: the detectors, the pulsed excitation and the electronic components. The IRF component from detectors (mainly APD) is less than 300 ps, from electronic components is less than 10 ps, and from excitation pulsed laser is less than 50 ps.[1] The IRF was measured using a pure clean glass substrate as a sample and the spectral filter was removed. We obtained an IRF of about 0.63 ns.

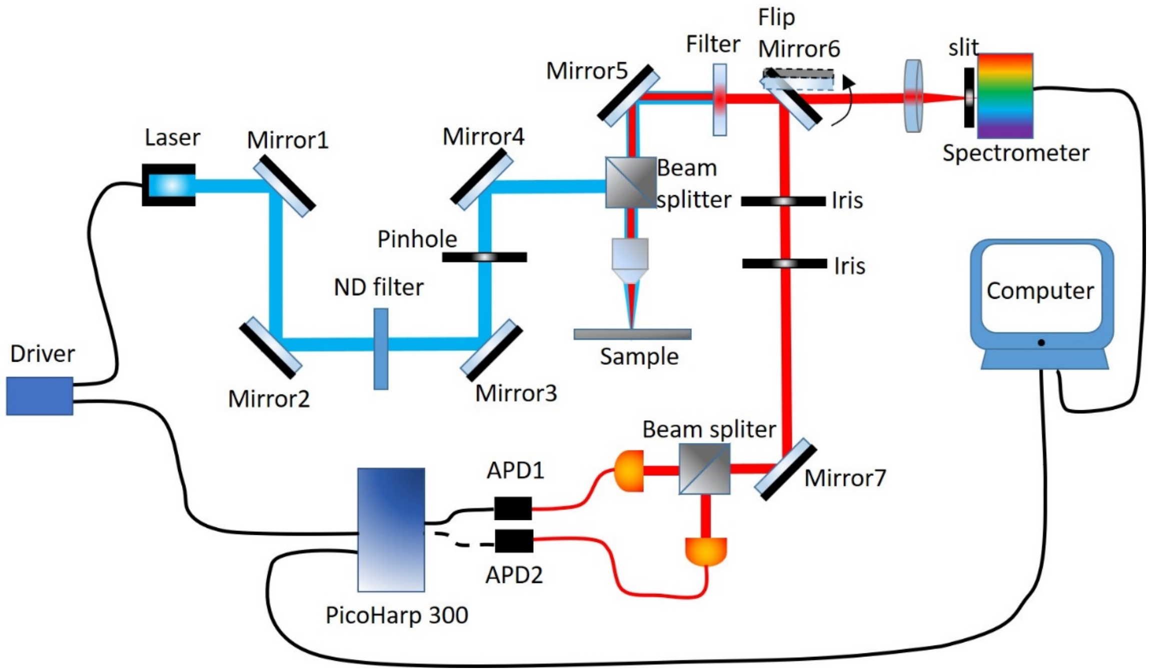

Supplementary Fig. 8 Schematic diagram of the experimental set-up used for life time and $g^{(2)}$ measurements.

In order to compare the emission properties of QDs in polymer with emission from QDs inside the polymer lobes of hybrid plasmonic nano-emitters (i.e. in the vicinity of Au nanostructures), single CdSe/CdS/Zn QDs embedded in polymer on glass substrate were analyzed. We used the low concentration photosensitive QD-containing formulation as we used for getting single-photon nanocube-based hybrid objects (see the end of the article). Polymer dots were fabricated on a clean glass substrate by 2-photon polymerization with an incident dose above the threshold. Decreasing the incident dose step by step led to polymer dots smaller than 500 nm and containing single QDs.

Supplementary Figure 9a shows the time evolution of the PL spectrum of six different QDs in polymer. A clear blinking is characteristic to single QD emission. Supplementary Figure 9b shows the measurement of lifetime and $g^{(2)}$ from three different single QDs embedded within sub-micronic polymer dots on glass substrate. It shows the signature of single photon emission ($g^{(2)}(0)$ in the 0.2-0.35 range) with a stable lifetime in the 17.5 ns - 17.9 ns range.

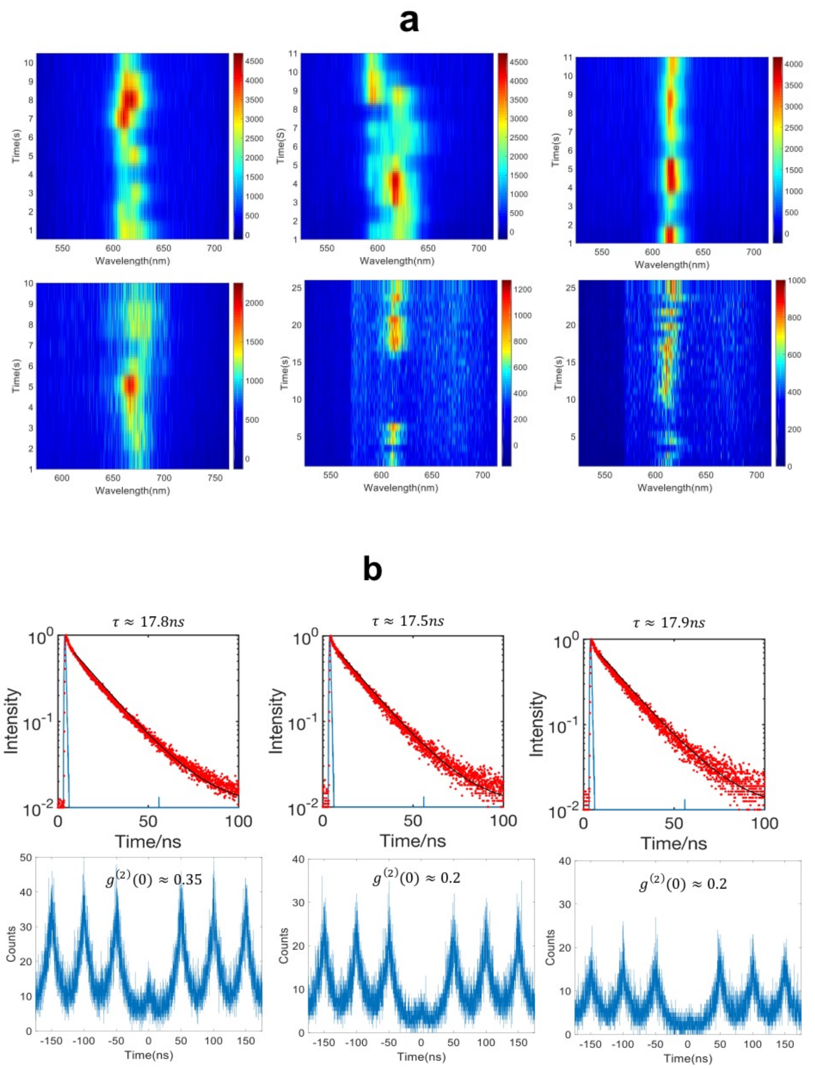

Supplementary Fig. 9 Emission properties of single QDs embedded in polymer dots on glass substrate. (**a)** time evolution of the PL spectrum from six different QDs (405-nm excitation wavelength). (**b)** lifetime (top, 0.62 μW.μm^{-2} incident power, 5 MHz repetition rate, the blue plot corresponds to the instrument response function and $g^{(2)}$ autocorrelation function (bottom, 2.5 μW.μm^{-2} incident power, 20 MHz repetition rate) measured from three different single QDs.

Supplementary Note 9. Life time measurements on different nanocube-based hybrid emitters containing single QD

Supplementary Figure 10 shows the photoluminescence lifetime measurement of three different hybrid nano-emitters similar to that of Fig. 3 of the manuscript, each of them contains single QDs. While lifetime of QDs in polymer dots on glass substrate is ~17.5 ns (see red plot on the right-hand side of Supplementary Fig. 10) life time of single QDs in

hybrid plasmonic nano-emitters varies in the 0.63 ns - 0.87 ns range (see left hand side of Supplementary Fig. 10), where 0.63 ns corresponds to the minimum life time our system can accurately measure at the time being.

This lifetime variation is attributed to the random position of the QD within the polymer lobes in the vicinity of the gold nanocube. It should be pointed out that some single-QD hybrid emitters showed very small lifetimes, close to the resolution of our system (~0.63 ns, see for example yellow curve, on the right-hand side of Supplementary Fig. 10) and even below, suggesting higher Purcell factor. These nano-emitters will be further studied, using the fact that, in principle, lifetimes down to 1/10 of the IRF width (FWHM) can still be recovered *via* iterative reconvolution.[1]

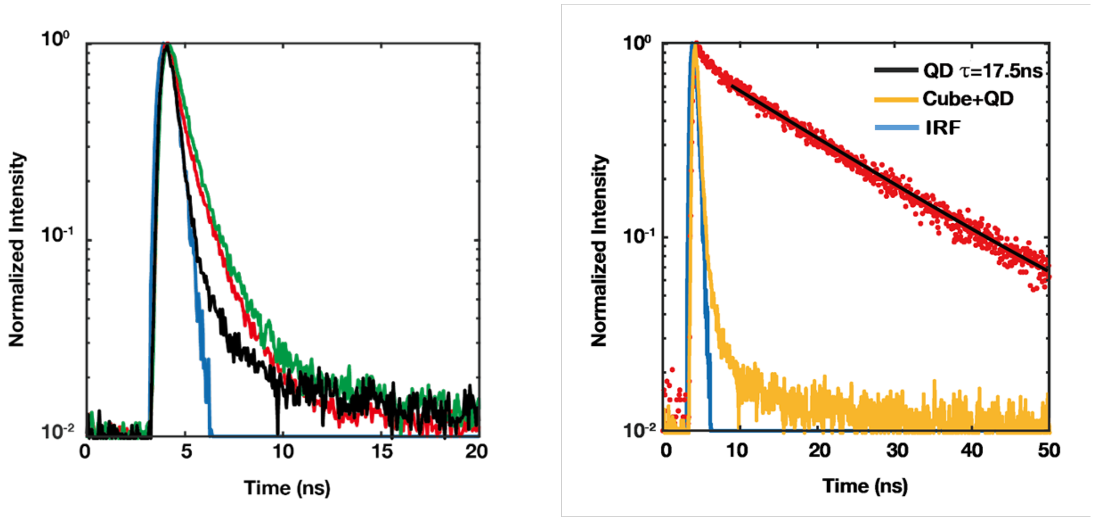

Supplementary Fig. 10 Left side: lifetime from three different nanocube-based hybrid emitters containing single QDs (0.62 μW.μm^{-2} incident power, 5 MHz repetition rate). Right side: comparisons with QDs embedded in polymer (red plots and black fitting), the yellow one represents the smallest measured lifetime from nanocube-based hybrid nanoemitters. In both figures, the blue curve represents the Instrument Response Function (IRF).

Supplementary reference

[1]Michael Wahl. “Time-Correlated Single Photon Counting”. PicoQuant Technical note. Available at: https://www.picoquant.com/images/uploads/page/files/7253/technote_tcspc.pdf
